# Correction to: Early Treatment of COVID-19 Disease: A Missed Opportunity

**DOI:** 10.1007/s40121-020-00366-7

**Published:** 2020-11-09

**Authors:** Jamie I. Forrest, Craig R. Rayner, Jay J. H. Park, Edward J. Mills

**Affiliations:** 1 Cytel Canada Health Inc., Vancouver, BC Canada; 2 Faculty of Health Sciences, McMaster University, Hamilton, ON Canada; 3 Certara, NJ USA; 4 Faculty of Medicine, University of British Columbia, Vancouver, BC Canada; 5 Monash Institute of Pharmaceutical Sciences, Monash University, Melbourne, VIC Australia

## Correction to: Infect Dis Ther 10.1007/s40121-020-00349-8

This article was originally published with the incorrect copyright license. The correct license should be CCBY not CCBY-NC. This has been corrected here.

